# Monkeypox Virus in Animals: Current Knowledge of Viral Transmission and Pathogenesis in Wild Animal Reservoirs and Captive Animal Models

**DOI:** 10.3390/v15040905

**Published:** 2023-03-31

**Authors:** Elizabeth A. Falendysz, Juan G. Lopera, Tonie E. Rocke, Jorge E. Osorio

**Affiliations:** 1 U.S. Geological Survey, National Wildlife Health Center, Madison, WI 53711, USA; 2 EDAN Diagnostics Inc., Madison, WI 53719, USA; 3 Department of Pathobiological Sciences, School of Veterinary Medicine, University of Wisconsin, Madison, WI 53706, USA; 4 Global Health Institute, University of Wisconsin, Madison, WI 53706, USA

**Keywords:** mpox, monkeypox, MPXV, wildlife, zoonosis, orthopoxvirus

## Abstract

Mpox, formerly called monkeypox, is now the most serious orthopoxvirus (OPXV) infection in humans. This zoonotic disease has been gradually re-emerging in humans with an increasing frequency of cases found in endemic areas, as well as an escalating frequency and size of epidemics outside of endemic areas in Africa. Currently, the largest known mpox epidemic is spreading throughout the world, with over 85,650 cases to date, mostly in Europe and North America. These increased endemic cases and epidemics are likely driven primarily by decreasing global immunity to OPXVs, along with other possible causes. The current unprecedented global outbreak of mpox has demonstrated higher numbers of human cases and greater human-to-human transmission than previously documented, necessitating an urgent need to better understand this disease in humans and animals. Monkeypox virus (MPXV) infections in animals, both naturally occurring and experimental, have provided critical information about the routes of transmission; the viral pathogenicity factors; the methods of control, such as vaccination and antivirals; the disease ecology in reservoir host species; and the conservation impacts on wildlife species. This review briefly described the epidemiology and transmission of MPXV between animals and humans and summarizes past studies on the ecology of MPXV in wild animals and experimental studies in captive animal models, with a focus on how animal infections have informed knowledge concerning various aspects of this pathogen. Knowledge gaps were highlighted in areas where future research, both in captive and free-ranging animals, could inform efforts to understand and control this disease in both humans and animals.

## 1. Mpox Epidemiology and Viral Transmission to Humans

Monkeypox virus (MPXV) is a complex cytoplasmic double-stranded DNA virus, belonging to the genus *Orthopoxvirus* (OPXV), family *Poxviridae* [1,2]. OPXVs are a diverse group that includes pathogenic viruses of significance to public health and veterinary medicine and low pathogenic viruses that circulate undetected in wild animals [3,4,5]. The OPXV genus is subdivided into two main groups, Old World OPXV and North American OPXV [4]. The members of the Old World OPXV group such as variola virus (VARV), the cause of smallpox in humans; vaccinia virus (VACV); cowpox virus (CPXV); and monkeypox virus (MPXV), are associated with human infection and can cause disease and death in humans [3,5]. Within OPXVs that infect humans, MPXV and VARV are the most virulent pathogens [6,7,8,9]. Due to their high pathogenicity in humans and environmental stability, both MPXV and VARV are considered potential bioweapons [10,11]. MPXV and VARV are biosafety level 3 and 4 agents, respectively, with restrictions for laboratory experimentation and use [10,12]. OPXVs are highly successful viruses known to infect a variety of mammalian hosts, especially rodents, with a broad geographic distribution [5,13]. Another relevant feature of members of the genus OPXV is their extensive serological cross-reactivity, which makes their unequivocal diagnosis dependent on more specific molecular techniques, such as PCR, DNA-sequencing, and viral isolation [14,15,16]. Due to the severe restrictions on VARV cultivation and research, other OPXVs, such as MPXV, have frequently been used as models to understand its virulence and pathogenesis [6,7,17].

MPXV is the causative agent of mpox disease in humans, a disease that resembles smallpox in its clinical manifestations [18,19]. MPXV infection can be fatal, with mortality rates as high as 10–17% for more virulent strains [6,20]. Because of the antigenic and genetic relationship between VACV, VARV, and MPXV, smallpox vaccines can protect against MPXV infection [7,21]. Human mpox is endemic in equatorial Africa, and, in contrast to the human-restricted infectivity of VARV, mpox is a zoonotic viral infection [9,19]. Mpox can be acquired by direct skin contact or mucosal exposure to infected animals, presumably rodents and monkeys [9,22]. Human-to-human transmission has often been mediated by close physical contact, but the airborne route has also been shown to play a role in secondary transmission [23]. Although human-to-human spread has historically been low, recent evidence has suggested that MPXV is becoming a more transmissible pathogen, and the recently recognized sexual transmission route could result in more efficient human-to-human transmission [24]. The incubation period is 10–14 days in humans, and the infectious period begins when clinical signs first appear, most commonly a pustular rash and lymphadenopathy [9,18,22].

Isolates of MPXV have been subdivided into two clades that have different geographic distributions and virulence [19,25,26]. The phylogenetic comparison of MPXVs has suggested a long temporal separation between clades. Natural human infection and experimental infection of animals with viruses isolated from Central Africa (clade I) have caused more severe disease, as compared to virus isolates from West Africa (clade II) [6,25,26,27]. Although early studies of mpox identified cases in both Central Africa and West Africa, from 1989 to 2013, human mpox cases were only reported in countries in Central Africa [24,28]. In the years prior (1970–1987), human cases occurred in Benin, Cote d’Ivoire, Gabon, Liberia, Nigeria, and Sierra Leone [24,28]. A serological survey in Ghana during the 2000s revealed the presence of antibodies against OPXV in humans and captured wild animals, suggesting the possibility of continued human contact with MPXV-infected animals [29], despite an apparent lack of human mpox cases in the region. Alternatively, a silent epizootic of an unknown OPXV could also explain these findings. Given this evidence and the sudden resurgence of human infections in the late 2010s, it was likely that MPXV was still circulating in animals and humans in regions of West Africa, even though human cases were not reported.

In 2003, MPXV was reported outside of Africa for the first time, after being introduced to the United States through the importation of infected rodents from Ghana [25,30,31]. The virus was transmitted to pet prairie dogs (*Cynomys ludovicianus*) co-housed with the imported African rodents and, subsequently, to humans [28]. Forty-seven human cases were reported in Wisconsin and Illinois, in persons who were in contact with the prairie dogs, but no cases of human-to-human transmission or deaths were reported [9,31]. A genomic sequence analysis showed that the virus belonged to clade II [28,31], making the outbreak in the United States the first incidence of human mpox originating from West Africa in over 20 years.

Until very recently, most reported cases of human mpox had arisen in Central Africa. Outbreaks of mpox have predominantly occurred in the Democratic Republic of Congo (DRC; formerly Zaire), where human cases have been reported since 1970 [18]. From 2007 to 2015, the DRC reported over 1000 cases per year, the highest in any country [32]. Cases in other Central African countries, including Cameroon, the Central African Republic (CAR), and the Republic of Congo (ROC) were intermittent, and outbreaks were usually small [24]. In 2005, human mpox cases were also identified in Sudan and were thought to be associated with possible translocations of infected humans or animals from Central Africa [33].

Beginning in the latter half of the 2010s, cases of mpox increased in both Central and West Africa. The majority of these cases occurred in the DRC, but numbers ranged from 2500 to 6216 per year in 2016–2021 [24]. In 2017 and 2018, a large outbreak of mpox occurred in Nigeria, with over 200 confirmed and suspected cases distributed over at least 17 states [34]. The distribution of cases in 2017 and early 2018 suggested multiple introductions from animals, as well as human-to-human transmission. This was the first confirmed human-to-human transmission of clade II MPXV [34]. During this outbreak, multiple exportation events occurred, to the United Kingdom (UK) [35], Singapore [36], Israel [37], and the United States [38]. These exported cases were primarily travelers who had visited Nigeria and healthcare workers [39]. Human mpox also increased during the period from 2015 to 2022 in Cameroon, the CAR, Liberia, the ROC, and Sierra Leone [24].

Since May 2022, the largest ever outbreak of mpox has occurred, affecting 102 countries outside of the endemic region, resulting in over 85,650 cases to date [40]. The virus isolates circulating in Portugal and Belgium in May 2022 were most closely related to the isolates from the 2018 cases of mpox exported from Nigeria to Singapore, the UK, and Israel, as well as travel-associated cases from Nigeria in 2021 [41], suggesting that the current multinational outbreak originated in Nigeria, similar to the 2017 exportation events. However, others [42] have suggested a more complex scenario indicative of “cryptic”, uncharacterized human-to-human transmission prior to the recent outbreaks and involving multiple countries. Viral genomes with a distinct lineage (A.2) have been found in three countries (United States, India, and Thailand) and appear to be linked to travel to Nigeria and the United Arab Emirates. This lineage is different from the B.1 lineage of the virus that was linked to recent cases in Europe. Therefore, several events may have led to the transmission of the virus outside Africa, with some most likely occurring before 2022 [43]. 

Clearly, mpox is an emerging disease. Despite a lack of reported human cases in West Africa from the mid-1980s to the mid-2010s, clade IIb MPXV from this region initiated a worldwide epidemic in 2022. An apparent shift has occurred since 2015, with much greater case numbers and, potentially, an increase in human-to-human transmission, of both the clade I and clade II strains of the virus [44]. In the last five years, regular exportations of MPXV from Africa to other countries have occurred, making it imperative to determine the genetic diversity of the viral strains involved and their potential pathogenicity, so public health officials can remain vigilant for changes that would further increase transmission or pathogenicity and lead to the world’s next pandemic. The recent work by Gigante et al. [45] indicated that the human apolipoprotein B editing complex (APOBEC3) cytosine deaminase may be driving the evolution of clade IIb viruses. It is currently unknown how genetic changes described in the virus strains isolated in the recent outbreak affect their transmissibility.

A decrease in the global immunity to OPXV since the cessation of smallpox vaccination campaigns has also been proposed as a potential reason for the increasing cases of mpox [22,46], as well as increases in international travel and spread across particular social networks [43]. Other potential causes are changes in the population and behavior of the reservoir species, increased hunting of wild animal reservoirs because of food insecurity and civil unrest, genetic changes in the virus, and improvements in the detection of mpox. Increased knowledge of the ecology of the virus in its natural host(s) is needed to address the root causes of the increased incidence and to develop public health solutions for this problem.

One potential solution is the development of improved medical countermeasures for mpox. Although two effective vaccines against MPXV are available (ACAM200, IMVAMUNE/JYNNEOS), a large part of the world population has no immunity against this pathogen, and the supplies of these vaccines are insufficient to quickly vaccinate large portions of the world population. Several contraindications related to the ACAM2000 vaccine, including immunosuppression, pregnancy, breastfeeding, heart disease, and atopic dermatitis [22,28,47], have complicated its use. The Imvamune/JYNNEOS vaccine has been approved for emergency use but is largely untested for efficacy in humans [48]. Additionally, two antiviral drugs (tecovirimat, brincidofovir) are available, but they have not been well-studied for the treatment of human mpox and have not been approved for widespread use globally [49,50,51,52,53]. The increase in genomic variability and changes in the mpox eco-epidemiology have raised questions about the possibility of MPXV becoming a highly transmissible human pathogen, similar to VARV, due to the long-term absence of vaccination [5,22,28,54,55]. The higher susceptibility of the human population, the possibility of continued MPXV spread into non-endemic regions, and its potential use as a bioweapon, highlight the need to study the mechanisms of infection, the virulence factors, and the potential drug targets, and to characterize the pathogenesis of MPXV in animal models [9,22,28,56]. Later in this review, we describe the currently known animal models that could be used to address these gaps.

## 2. Monkeypox Virus in Wild Animals

Mpox is a zoonosis, but much remains to be understood about its ecology. MPXV can infect a wide variety of mammalian species, and rodents are believed to be the most likely reservoir of the virus [57], though primates have also been considered as possible reservoirs. Despite many years of epidemiological surveillance for MPXV, the specific reservoir species of the virus have not been definitively identified [9,18]. This presents a major barrier to understanding the ecology of this virus, and one that deserves urgent attention. Furthermore, the geographic separation of clade I and clade II viruses has indicated the possibility that the reservoir(s) could also be distinct for each clade.

MPXV was named after being isolated from laboratory monkeys in 1958 (von Magnus et al. 1959). Despite this, early serosurveys indicated the virus did not seem to be widely circulating in primate species [46]. MPXV has only been isolated directly in wild animals on rare occasions: from a Thomas’s rope squirrel (*Funisciurus anerythrus*) in the DRC in 1978, from a sooty mangabey (*Cercocebus arys*) in Cote d’Ivoire in 2010, and most recently, in a troop of chimpanzees (*Pan troglodytes*) in Cote d’Ivoire in 2017 [58,59,60]. The serological evidence of infection in wild animals has been found most often in rodents. In 1987, a very large investigation of MPXV reservoirs found that 24.7% of Thomas’s rope squirrels were seropositive in Zaire (now the DRC) [59]. The outbreak of mpox in the United States in 2003 was associated with the importation of pouched rats (*Cricetomys* spp.), rope squirrels (*Funisciurus* spp.), and African dormice (*Graphiurus* spp.), from Ghana [31]. In a 2004 follow-up investigation in Ghana, OPXV antibodies were found in pouched rats, African dormice, rope squirrels, and sun squirrels (*Heliosciurus* spp.), and OPXV DNA was found in the tissues of pouched rats, African dormice, and African ground squirrels (*Xerus* spp.) [29]. Neither of these results was specific for MPXV, so they could be evidence for MPXV circulation, or one or more other OPXVs could be circulating in these animals. A more recent study in Nigeria found that OPXV antibodies were found in *Praomys* spp. and *Rattus rattus*. Though there had been recent human mpox cases in Nigeria at the time, another rodent in the study (*Mus baoulei*) was found to be PCR-positive for OPXV but not MPXV, suggesting the circulation of another poxvirus in the local rodent populations [61]. Following an investigation of human mpox in 2017 in the Republic of Congo (ROC), 22% of Emin’s pouched rats (*Cricetomys emini*) were found to be seropositive [62]. Conversely, in another study, the same investigators found that mpox was epidemiologically linked to contact with non-human primates, and not rodents, in the DRC [63]. Lastly, MPXV-viral DNA was found in 93 of 1038 (9.0%) of the museum specimens of five *Funisciurus* species (*F. anerythrus*, *F. carruthersi*, *F. congicus*, *F. lemiscatus*, *and F. pyrropus*) in Central Africa [64]. 

Captive infection studies have also contributed to our understanding of MPXV in potential reservoir hosts from endemic areas in Africa. Several groups have performed experimental infection studies to characterize MPXV infection in Gambian pouched rats (*Cricetomys gambianus*) and rope squirrels (*Funisciurus* spp.). Mortality and morbidity were higher in rope squirrels than in pouched rats, but both species demonstrated some viral replication via in vivo bioluminescent imaging (BLI), in the absence of clinical signs [65,66]. It was also clear from these studies that MPXV could remain persistent in infected tissues for several weeks in both pouched rats and rope squirrels [65,66,67]. These animals shed high titers of MPXV in oral, nasal, and rectal secretions, which could have led to environmental contamination with MPXV and potential transmission to humans. An African dormouse species, Kellen’s dormouse (*G. kelleni*), was shown to be highly susceptible to intranasal (IN) infection with clade I MPXV, with an IN LD50 of only 12 pfu. The animals infected with 2 × 10^4^ pfu shed the virus in nasal washes as early as day 2, indicating this species likely would have shed infective virus into the environment by nasal secretions [68]. In a separate study, in vivo imaging also demonstrated that the virus had been replicating in the nasal area of infected dormice as early as five days post-infection, although it was not clear from the report whether clinical signs had been observed during this early period of viral replication [69]. Currently, we are characterizing the infection of additional potential wild reservoir species captured in the DRC: Emin’s pouched rats (*Cricetomys emini*) and thicket rats (*Grammomys surdaster*). Further animal work could elucidate the most likely routes of transmission to help understand the length of time during which these species may be able to maintain and shed the virus and to model maintenance and transmission of MPXV in various species. This work could also parallel other work in the field to better understand the ecology and social behavior of these host species and how humans interact with them.

The practical application of this information about native host species could also include educational campaigns to consumers of bush meat in Africa. For example, some species, such as primates, may only be infectious when they have skin lesions, whereas Gambian pouched rats had infectious MPXV in their tissues without gross evidence of disease [65]. This information could inform mitigation strategies, such as avoiding consumption of certain species or the use of gloves when handling uncooked specimens of certain species. As third-generation smallpox vaccines become more widely available, people that hunt and prepare bush meat, including rodent and primate species, could be prioritized for vaccination, along with healthcare workers.

## 3. Animal Models Used to Study the Monkeypox Virus 

Captive animal studies have been used to understand many factors about MPXV infection in humans and how we could treat and prevent infections. Since the 1980s, researchers have searched for an animal model of mpox that mirrored the symptoms of human infection and that could be used to assess mpox pathogenesis as well as variations in viral virulence factors, and to test antiviral drugs and vaccines. To date, no perfect animal model has been identified, but several have been useful, with various positive and negative aspects. 

Early studies assessed the susceptibility of several rodent species to MPXV, including cotton rats (*Sigmodon hispidus)*, red squirrels (*Sciurus vulgaris*), rabbits (*Oryctolagus cuniculus*), white rats (*Rattus norvegicus domestica*), and white mice (*Mus musculus*) [27,70,71]. These studies showed that the susceptibility of rodents to MPXV infection varied with the inoculation route, and reduced susceptibility was associated with age. Adult rodents tended to be more resistant to MPXV infection, while newborn animals were highly susceptible when inoculated by various routes, resulting in high morbidity and mortality rates. Because of the small size of the animals and the immature status of their immune systems, infant rodents are not currently used as animal models of MPXV infection [27]. Additionally, these models did not completely mimic human mpox clinical presentation and pathogenesis [13,71,72]. More recent experiments confirmed that the chinchilla strain of laboratory rabbits and Siberian miniature pigs (*Sus scrofa*) were not susceptible to infection by MPXV [73].

MPXV infection has also been studied experimentally in nonhuman primates, and primate models have been used to test vaccines and other medical countermeasures [6,13,23,72,74]. Rhesus macaques (*Macaca mulatta)* developed severe fatal disease between 7 and 14 days, and the infection was characterized by maculopapular pox lesions in the skin and mucous membranes of the oral cavity, as well as hemorrhages in multiple organs and lymphadenopathy [75,76,77,78]. Cynomolgus monkeys (*Macaca fascicularis*) were highly susceptible to infection and showed clinical signs that resembled human mpox, including vesiculopapular rash, fibrinonecrotic pneumonia, lymphadenopathy, and death within 9–17 days post-infection [79,80]. Both models have typically been used with a lethal dose of approximately 5 × 10^7^ PFU by the intravenous (IV) route [81]. Both intranasal and aerosol exposures of MPXV in cynomolgus macaques yielded similar results [23,76,82]. Marmosets have also been suggested as a potential model for human mpox, but their signs of the disease more resemble smallpox in humans, rather than mpox [83]. When given IV, moderate doses led to petechiae, pronounced lethargy, and death within three days, resembling the hemorrhagic form of smallpox disease. Lower doses adequately reproduced the lymphadenopathy found in human mpox, but the skin lesions did not progress through the typical OPXV lesion stages. The pulmonary lesions were less consistent than in the cynomolgus macaque model. In marmosets infected IN, typical OPXV skin lesions were seen in 2 of 4 marmosets. Dyspnea was evident, but the histology from the lungs and other organs was not reported. Large size, high cost, and the desirability of using lesssentient animal models have been limiting factors in primate models. Table 1 compares multiple primate and rodent models of MPXV infection by route, dose, and viral clade. 

The emergence of MPXV in the United States in 2003, following the importation of infected rodents from Africa, raised the possibility of using wild animals as models for mpox [13,72]. Three African rodent species were identified as potential sources of MPXV in the 2003 outbreak: rope squirrels (*Funisciurus* sp.), Gambian giant pouched rats (*Cricetomys gambianus*), and dormice (*Graphiurus* sp.) [31]. In laboratory studies, MPXV infection of dormice (*Graphiurus kelleni*) was highly lethal; the animals died within 7–8 days after IN infection, but no differences in morbidity and mortality were observed between MPXV clades [68]. This animal model was used to validate the prophylactic and therapeutic uses of smallpox vaccines against MPXV [68]. 

The 2003 MPXV outbreak in the United States also revealed a new potential animal model of mpox, as black–tailed prairie dogs (*Cynomys ludovicianus*) that had contact with infected African species were inadvertently infected, became sick, and died during that outbreak. The prairie dog model resembled the clinical characteristics of the human mpox disease and has been used extensively as a model to study the pathogenesis and transmission [13,72]. Black-tailed prairie dogs infected via intraperitoneal (IP), intradermal (ID), or IN routes developed severe lesions and clear signs of mpox. Systemic viral replication (blood, spleen, lungs, skin, liver, kidney, and heart) was detected after six days post-inoculation. Severely affected animals developed skin, tongue, and lip lesions and died within 8–16 days. These studies confirmed high levels of MPXV shedding via oral, nasal, ocular, and rectal routes, as well as animal-to-animal oral, respiratory, and mucosal transmission [87,90,91]. Because of the similarity to human mpox, prairie dogs have been used for vaccination and antiviral studies for the prevention and treatment of mpox [13,72,92]. Other species of ground squirrels may be similarly susceptible to MPXV and may be easier to acquire in some areas than black-tailed prairie dogs. Sergeev et al. [88] found that the Bobak Marmot (*Marmota bobak*) displayed similar lesions and tissue tropism to black-tailed prairie dogs, making them another potentially useful animal model of mpox.

Other wild rodents from North America have also been used to study the MPXV pathogenesis and transmission [13,72,87,90,91,93]. The experimental infection of 13-lined ground squirrels (*Spermophilus tridecemlineatus*) by the IP route resulted in clinical disease and death within 7 days, whereas animals infected via the IN route died by day 9 post-infection [93]. All animals became lethargic and anorexic. However, neither detectable skin lesions nor respiratory symptoms were observed. Following an IP injection, the virus was detected in the blood by day 3 and throat swabs by day 4, with peak titers by day 5. Independent of the route of infection, high virus titers were found in the liver and spleen, but less in the kidney, lung, and heart [93]. Due to the lack of clinical signs compatible with human infection, the utility of this model is limited. 

Although wild animals have proven helpful in the study of MPXV pathogenesis, their use has been restricted by seasonal and poor availability, a lack of species-specific reagents, and difficult reproduction in captivity. Therefore, a more cost effective and easily produced animal model to study MPXV infection is needed [27]. Several laboratory mouse strains have been evaluated for their potential to characterize the replication and virulence differences between MPXV clades. However, most mouse strains have been resistant to infection, and differences in mortality were not observed [27,86,94]. In common laboratory mouse strains, such as BALB/c, C57BL/6J, as well as many others, high doses of MPXV inoculation did not cause mortality, and differences in morbidity between viral clades were difficult to identify [84]. Even the lack of type I and II interferon receptor genes in AG129 mice did not make them susceptible to disease from infection with clade II MPXV. Neither IN nor IP infection caused clinical signs of MPXV infection (File S1), but the virus replicated for longer periods (Figures S1–S6), up to 30 days post-inoculation [95]. SCID BALB/c mice were highly susceptible to IP MPXV infection, and the time-to-death was longer for clade II MPXV than clade I [86]. 

### 3.1. Animal Studies on Immune Responses to Monkeypox Virus 

One specific knowledge gap that could be filled using data from animal studies is how MPXV interacts with the immune system. This is an important topic because the differences in the pathogenicity of the two clades of MPXV appear to be related to the presence of immunomodulatory genes in clade I viruses that are not present in clade II viruses [96]. Additionally, the development of improved vaccines requires the advanced study of the immune response following vaccination in an animal model. Much work has been performed using non-human primate models to understand the antibody response and the T-cell response to MPXV and mpox vaccines in cynomolgus and rhesus macaques [78,80,97,98]. Others have also used macaque models to study how antiviral medications co-administered with vaccines affect immune responses [81,99].

To further this work, an immunocompetent mouse model that is susceptible to MPXV is needed. Sergeev et al. showed that IN infection with high doses of MPXV in outbred ICR mice caused pulmonary disease that was reduced with antiviral drugs. However, this model did not result in mortality, and the outward signs of clinical disease were limited to ocular disease and ruffled fur [73]. Although the severe pathology that could lead to mortality and the skin lesions typically associated with mpox were not evident in the ICR mice, this could be considered an immune-competent mouse model of viral pneumonia caused by MPXV. 

To date, the most common mouse breed used as a model of mpox has been the CAST/EiJ mouse. This mouse model was derived from *Mus musculus castaneous* and has been shown to be highly susceptible to MPXV [100]. The mouse-specific reagents available for studying the immune function of other strains of *Mus musculus* have worked well in this strain. The CAST/EiJ mouse model also has several unique features, as compared to other laboratory mice. MPXV infection in CAST/EIJ mice is lethal in a dose-dependent manner, and adult animals are susceptible to infection. Viral replication and spread caused systemic infection (lymph nodes, lung, spleen, liver, heart, and kidneys), significant weight loss, and mortality at days 6–10 post inoculation. More importantly, the immunization of CAST/EiJ mice with VACV induced antigen-specific T- and B-lymphocyte responses that protected the mice from lethal doses of MPXV, which supports the immune competence of this model [84,100]. Previous studies of CAST/EiJ mice concluded that the reason for their MPXV susceptibility was due to a deficiency in the IFN-γ production in the lung. However, they also showed that the levels of interferon in the other organs, including the spleen, were similar or greater than those measured in BALB/c mice [85]. This was evidence that CAST/EiJ mice are not lacking the IFN-γ gene or its expression, and their susceptibility is likely due to a lower level of circulating natural killer (NK) cells, which are major producers of interferons [101]. Later work by Earl et al. confirmed that CAST/EiJ mice had lower numbers of circulating NK cells and that this could be overcome with either a treatment of IL-15 or with a passive transfer of additional NK cells [102]. Therefore, although there is a difference in the innate immunity of the CAST/EiJ mice that makes them susceptible to OPXV, including MPXV, they can be considered immunocompetent. This model was recently used to confirm the efficacy of the antiviral drug tecovirimat for the MPXV strain circulating in the recent 2022 outbreak [103].

### 3.2. The Utility of In Vivo Imaging in Investigation of Monkeypox Virus Pathogenesis

Based on the limitations of the various animal models for studying human mpox, it has become increasingly clear that a more thorough characterization of the disease with real-time technologies would be valuable [104,105,106]. Additionally, conventional pathogenic studies have required the sacrifice of numerous infected animals, which has limited MPXV experimentation [107]. In vivo imaging is a useful tool that could help resolve both concerns. In vivo imaging has been used extensively to detect fluorescent and luminescent signals in live animals in a variety of studies [104,105,106,108,109]. These signals have been coupled with pathogens (virus, bacteria, or protozoa), antibodies, and other biomolecules to study their presence, amount, and distribution inside live animals over time [106,107]. Bioluminescent imaging (BLI) refers specifically to in vivo imaging using luminescence detection. This methodology has been used to study and characterize pathogen infection and to evaluate the host immune-signaling pathways, cell-trafficking, and tumor growth, enabling the real-time quantification and analysis of experimental treatments [104,105,106,107].

Taking advantage of the capacity to stably incorporate foreign genes into OPXV genomes [110,111], the insertion of luciferase and fluorescent genes markers has been used repeatedly to compare the infections of MPXV, CPXV, and VACV in rodents and primates [69,86,90,100,107,108]. Studies have shown that the insertion of the luciferase marker did not alter the viral replication or attenuate the virus, in vitro or in vivo, providing significant advantages for studying viral and host factors to determine the pathogenesis [86,106,112]. For example, Luker et al. [112] showed that the replication and the tissue dissemination of VACV was significantly increased in mice lacking receptors of type I and II IFNs, as compared to wild-type mice. In the same study, the authors demonstrated that the focal tissue luminescence was directly proportional to the virus titer in the tissue, enabling the quantification of the relative amounts of virus in various anatomical sites by BLI [112]. In our own study, BLI was used to show the differential replication of the clade I and II MPXV strains, despite a lack of differences in morbidity and mortality [86]. Using the same methodology to study MPXV infection, Earl et al. [69] constructed another recombinant clade I MPXV isolate expressing the luciferase gene and compared the outcome of IN infection of MPXV on the virulence, the tissue tropism, and the kinetics of replication between susceptible (CAST/EiJ) and resistant (BALB/c) mice strains. This study was conducted in two mouse models, and it also showed that the dissemination and the replication of the recombinant OPXV/Luc+ could be tracked within the internal organs. However, the analysis of the individual tissues from the CAST/EiJ mice showed that the luciferase emissions in different organs and body regions of mice infected with MPXV/Luc+ were not always directly associated with the tissue titers [69]. For example, when the luminescence in the area of the lung was equal to the luminescence in the abdomen, the viral titers in the lung were much higher than the titers in the liver and spleen. Similar findings were also found in the large prairie dog model, which were likely exacerbated by the larger amount of tissue between the tissues of origin and the imaging camera [90]. 

BLI has also been used to study interactions between the MPXV-elicited immune responses and the replication of the virus in vivo. We used CAST/EiJ mice and BLI to demonstrate that deletion of viral genes for secreted inhibitors of type I and type II interferons, an inhibitor of IL-1β, and two apoptosis modulators decreased viral pathogenicity and viral replication in mice but did not reduce viral replication in cell culture [96]. Future work could elucidate the effect of deletion of these genes individually. Earl et al. [102] used BLI to demonstrate that the addition of NK cells and IL-15 was sufficient to overcome the susceptibility of CAST/EiJ mice to VACV. Other examples of the use of BLI in OPXV research were in the preclinical evaluation of smallpox vaccines and the efficacy studies of antiviral treatments [113,114]. This technique has also been used to evaluate the CAST/EiJ mouse model for VACV and CPXV infections, vaccine testing, and the development of antiviral drugs [100,103]. Altogether, these studies using BLI demonstrated the utility of this methodology to compare and track MPXV and OPXV infections in mouse models, to evaluate host susceptibility, and to study the efficacy of vaccines and antivirals for mpox [86,100,112]. 

Not only is BLI a useful tool for understanding the virulence of MPXV strains, but it also has the potential to fulfill critical needs in the study of mpox epidemiology and pathogenesis in wild rodent species. We used BLI to track MPXV infection in potential reservoir species, such as rope squirrels and pouched rats, enabling us to monitor in real time where the virus was replicating in high amounts (the oral and nasal cavities) [65,66]. Using this method, we discovered viral replication at sites in the skin, without visible lesions in Gambian pouched rats [65], demonstrating that BLI could detect viral presence in areas that would not normally be collected for viral detection by PCR or viral culture. 

### 3.3. Investigations of MPXV Genetic Diversity in Relation to Pathogenesis and Virulence

Recent findings have shown that the genetic diversity of MPXV in Central Africa is increasing; however, little is known about the differences in pathogenicity and the host range of circulating MPXVs. Sequencing and phylogenetic analyses have shown five short branches (lineages) within clade I viruses, suggesting a more recent diversification that could reflect viral adaptation to different hosts. In addition, the distribution of these viruses showed some associations between geographic origin and viral genetic variation. The genomic comparison of these circulating lineages showed genomic regions with high variability that could be associated with differences in the virulence and transmission in different reservoirs and humans [33,54,55]. Future work could investigate whether genomic differences between these five MPXV genotypes could explain the differences in pathogenicity. Using BLI in combination with the CAST/EiJ mouse model would be an ideal system for understanding the more subtle differences between the genotypes within a viral clade. Similarly, the methodology used in this research could be used to characterize the pathogenesis and transmissibility of the current clade II outbreak strains of MPXV and closely related viruses circulating in West Africa. 

Multiple studies showed that the CAST/EiJ animal model was useful for studying MPXV virulence factors, as well as the host immune response [96,102]. This model could be used in future studies to determine how viral genetics influence immunomodulation and transmission and if these genetic components are changing over time with increasing human-to-human transmission. On the other hand, prairie dogs have displayed clinical signs that more closely resemble mpox in humans. To understand the role of the virulence genes in the pathogenesis, future studies could use the prairie dog model to study the effects of the immunomodulation genes on the clinical signs and the progression of MPXV infection. This model, in concert with BLI, could be a very useful tool for studying the mechanism behind the apparent increase in transmissibility in recent outbreaks. 

### 3.4. Alternative Routes of Monkeypox Virus Transmission 

The 2022 global outbreak of mpox revealed a potentially novel method of transmission: sexual contact. Sexual activity was highly common among human transmission events across the globe [57]. It is currently unknown if sexual contact is only an effective method of close contact with skin and mucous membranes or if sexual transmission via reproductive fluids such as semen is also possible. This determination would require studies of semen and testicular tissues, likely in larger animal models, such as primates and prairie dogs. The collection of semen over time would be most easily accomplished in primates. In the prairie dog model using BLI, viral replication was evident in the testicular tissues [90], and in SCID BALB/c mice, the virus and pox lesions were detected in the ovaries of the female mice [86]. A recent retrospective investigation uncovered MPXV in the testes of rhesus macaques for periods as long as 37 days post-exposure [115].

The reports of spontaneous early miscarriages, as well as a second-trimester stillborn fetus, in human females that contracted mpox were most likely related to a clade I strain [116], which has raised concerns about the vertical transmission of the virus and the outcomes of the infection in pregnant people [117]. The tissues of the stillborn fetus were positive for MPXV DNA. Studies in primate models with both viral clades would be helpful in evaluating the risks of MPXV infection on maternal and fetal morbidity and mortality, as well as potential treatment options. 

### 3.5. The Use of Animal Models to Develop Medical Countermeasures

Nonhuman primates, prairie dogs, and laboratory mouse models have been used to evaluate medical countermeasures, such as antiviral drugs and vaccines. For example, the oral administration of the antiviral drug, tecovirimat, was shown to protect primates from MPXV disease by significantly reducing viral loads, clinical signs, and mortality [118]. Similarly, mortality was significantly reduced in treated prairie dogs [119]. This drug was shown to be safe in numerous subsequent clinical trials in humans [57], although more work is needed to confirm its efficacy and usefulness for treating mpox patients. Another antiviral drug, brincidofovir, was also tested using the prairie dog model and shown to be most useful if administered early in the course of infection [53]. Although licensed by the U.S. Food and Drug Administration to treat human smallpox disease, more studies are needed on brincidofovir to determine its safety and efficacy in treating mpox patients. Animal models would be particularly useful for studying whether the development of drug-resistant strains of MPXV could occur with extended use. 

Potential vaccine candidates have also been screened and compared in animal models. For example, cynomolgus macaques that received a single immunization with the ACAM2000 attenuated smallpox vaccine were shown to be protected against aerosolized MPXV, whereas the newer third-generation vaccine, modified vaccinia Ankara (Imvamune/JYNNEOS), required an initial dose and a booster to elicit full protection [98]. In the prairie dog model, the vaccination with ACAM2000, 1–3 days after MPXV infection, significantly reduced mortality from infection, whereas Imvamune was only effective if administered 1 day post-exposure [92]. However, if administered prior to exposure, Imvamune elicited long-lasting protection against MPXV challenge, and because it is a non-replicating viral vaccine, it was considered safer, causing fewer side effects than ACAM2000 [120]. In these examples and others, the usefulness of screening new medical countermeasures in animal models is evident. The recent outbreak has stimulated a renewed interest in developing additional countermeasures to prevent and treat mpox, and several new products are currently being tested in animal models. 

## 4. Conclusions

Many questions remain about MPXV epidemiology that require animal studies to answer. The most prominent of these questions are: (1) What are the reservoir species of MPXVin the wild? (2) What is the basis (viral genetics, host behaviors, or others) for the apparent increase in transmissibility in recent cases of human mpox? (3) How does MPXV interact with the immune system? (4) Can we use the genetic basis of these interactions to predict which strains may be more likely to become epidemic strains in the future? This review demonstrated that several animal models may be helpful in answering these questions about the viral pathogenesis of MPXV, most importantly in CAST/EiJ mice. The prairie dog and cynomolgus monkey models represent the best options to further test the immunoprophylaxis and antiviral treatments. The prairie dog model also holds promise in studying the transmission of the various MPXV strains. The identification of likely MPXV reservoir species for both clades of the virus will require both laboratory-based captive studies and extensive field work. A better understanding of the epizootiology of MPXV will help health officials determine which populations of humans should be targeted for prevention strategies. As we have seen, MPXV will likely continue to emerge in humans without further intervention. Only through continued studies in animals can we answer some of these long-standing questions and start implementing appropriate public health strategies.

## Figures and Tables

**Table 1:** Outcomes of infection in animal models used for the study of mpox, by species, dose, and route of infection, including intranasal (IN), intraperitoneal (IP), intradermal (ID), subcutaneous (SC), and intravenous (IV).

Animal Model	Route	Dose (pfu)	Mortality	Clinical Signs	Lung Pathology	Skin and Mucous Membrane Pathology	Other Pathology	Clade Differences	Ref.
CAST/EiJ mouse	IN	1 × 10^4^ to 1 × 10^6^	100%	WL	NR	None	NR	Yes	[84]
		1 × 10^3^	60%						
		1 × 10^2^	0%						
	IP	100–1000	100%	WL, hunched posture, and ruffled fur,	NR	None	NR	Yes	[84,85]
		10	50%						
		1	0%						
ICR mice	IN	6.3 × 10^3^ to 1 × 10^5^	0%	purulent conjunctivitis, blepharitis, ruffled fur	pulmonary edema, necrotic tracheitis and bronchitis	NR	microvascular damage	NR	[73]
SCID Balb/C mouse	IP	1 × 10^5^	100%	rough coat, inappetence, LG	NR	intradermal bullae in the footpads	necrotic ovarian follicles, necrotic enteritis	Yes	[86]
African dormouse	ID	1.40 × 10^4^	92%	NR	NR	NR	NR	NR	[68]
	IN	0.2–2000	2000 pfu: 100% 200pfu: 100% 20 pfu: 63% 2 pfu: 38% 0.2 pfu: 0%	NR	NR	rhinitis with syncytial cell formation in nasal mucosal epithelium	NR		
black-tailed prairie dog	IN	6.00 × 10^5^	75%	LG, LAP, WL lethargy,	NR	VPR, ON	NR	Yes	[27]
	IN	1.25 × 10^6^	60%	LG, AR, nasal discharge	edema, hemorrhage, and necrosis of the lung	ON	mild lesions in spleen, liver, and adipose	NR	[87]
	IP	1.25 × 10^6^	100%	LG, AR	mild thickening of the pulmonary interstitium, plasmacytic infiltrates	NR	necrosis of adipose, liver and spleen; vasculitis	NR	[87]
Bobak’s marmot	IN	158 to 1.26 × 10^7^	25–100%	F, LAP, incoordination, aggression, blepharitis	FNP	VPR, necrotizing dermatitis	thrombohemorrhagic syndrome, splenic LD, thymic necrosis, necrotic lymphadenitis	NR	[88]
	SC	6300 to 1.26 × 10^7^	100%	F, LAP, incoordination, aggression, blepharitis	FNP	VPR	thymic necrosis, necrotic lymphadenitis	NR	[88]
Cynomolgus macaque	IV	5.00 × 10^6^	~70%	F, LAP, WL, LG, AR, nasal discharge, edema of hands, feet, and head	proliferative and necrotizing lesions of the trachea and lung, FNP	VPR, ON	splenic LD, hemorrhagic GI lesions, epicardial petechiae	NR	[79]
		5.00 × 10^7^	33–100%						[79,80]
	IN	1.00 × 10^6^	clade I: 75% clade II: 33%	F, WL, LG, AR, diarrhea	unspecified severe lung pathology in clade I infection, but not clade II.	VPR	lesions in GI genitourinary system, and reticuloendothelial organs	Yes	[82]
	Aerosol	clade I: 110–2 × 10^4^ clade II: 90–5 × 10^5^	clade I, 110 pfu: 0% clade I 20,000 pfu: 100% clade II: 0%	F, AR, cough, nasal discharge, depression, weakness	FNP	VPR, ON	GI lesions, lymphadenitis, splenitis	Yes	[23,76]
Rhesus macaque	IV	5.00 × 10^8^	100%	F	NR	prodromal rash	multi-organ hemorrhagic disease	NR	[80]
		5.00 × 10^7^	80–100%	F, LAP, WL, LG, AR, nasal discharge, edema of hands, feet, and head	pulmonary edema	VPR, ON	NR	NR	[77,78]
		5.00 × 10^6^	0%	F, LAP, coagulopathy	pulmonary hemorrhage	VPR	multi-organ hemorrhagic disease, hepatopathy, splenomegaly, bone marrow necrosis	NR	[89]
Common Marmoset	IV	48 to 2.4 × 10^7^	100%	LG, LAP, unkempt coat	inconsistent hemorrhage or edema in the lungs	erythematous and petechial rash	LD in spleen and lymph nodes; necrosis in spleen, lymph nodes, bone marrow, and adrenals; hepatic lesions	NR	[83]

NR = not reported. PFU = plaque-forming units. FNP = fibrinonecrotic pneumonia (including pleuropneumonia or bronchopneumonia). VPR = vesiculopapular rash (typical *Orthopoxvirus* rash). WL = weight loss. LAP = lymphadenopathy. LG = lethargy. F = fever. ON = Oronasal lesions, including on the oral mucosa, lips, nares, tongue, and esophagus. Ref = references. GI-gastrointestinal tract. LD = lymphoid depletion. AR = anorexia.

## Data Availability

Data for the supplementary material are available at Falendysz, E., Lopera, J., Rocke, T., and Osorio, J., 2023, Luminescence of AG129 mice infected with recombinant monkeypox virus expressing firefly luciferase: U.S. Geological Survey data release, https://doi.org/10.5066/P9S0ABHH (accessed on 24 March 2023).

## Supplementary Materials

The following supporting information can be downloaded at: https://www.mdpi.com/article/10.3390/v15040905/s1, File S1: Luminescence of AG129 mice infected with recombinant monkeypox virus expressing firefly luciferase [95]. Figure S1. Bioluminescent images of AG129 mice infected with MPXV/USA/Luc intraperitoneally. Figure S2. Bioluminescent images of AG129 mice infected with MPXV/USA/Luc intraperitoneally. Figure S3. Bioluminescent images of AG129 mice infected with MPXV/USA/Luc intranasally. Figure S4. Bioluminescent images of AG129 mice infected with MPXV/USA/Luc intraperitoneally. Figure S5. Bioluminescence measured as total flux in photons per second (p/s) of AG129 mice infected intraperitoneally with MPXV/USA/luc. Figure S6. Bioluminescence measured as total flux in photons per second (p/s) of AG129 mice infected intranasally with MPXV/USA/luc. References [86,95] are cited in the supplementary materials.
