# Supplementary material for: Monkeypox Virus in Animals: Current Knowledge of Viral Transmission and Pathogenesis in Wild Animal Reservoirs and Captive Animal Models
Source: Viruses. 2023 Mar 31;15(4):905. doi: 10.3390/v15040905 (PMC10142277; doi:10.3390/v15040905)
Supplement: Supplementary file 1 [file viruses-15-00905-s001.zip › viruses-2239772-supplementary.pdf]

Review

Monkeypox Virus in Animals: Current Knowledge of Viral Transmission and Pathogenesis in Wild Animal Reservoirs and Captive Animal Models

Supplement S1: Luminescence of AG129 mice infected with recombinant

Monkeypox virus expressing firefly luciferase.

Methods: Adult AG129 mice (129/Sv background deficient in alpha/beta interferon (IFN-α/β) and IFN-ɣ receptors) were obtained from a pathogen-free colony at the University of Wisconsin- School of Veterinary Medicine. The colony was established from mice provided by B&K Universal Limited (Hull, England). Immune-deficient mice were housed in hepa-filtered boxes, and provided an irradiated diet and acidified water. Experiments were conducted at the U.S. Geological Survey National Wildlife Health Center (NWHC), ABSL-3 tight isolation animal facility, under NWHC animal care and use protocol EP090616. Experimental infections were conducted with clade II Monkeypox virus (MPXV) that expresses firefly luciferase (luc), designated MPXV/USA/luc, as described in Osorio et. al [1]. Twelve AG129 mice (8 males and 4 females) were infected intranasally with 10^5 PFU (5 ul, each nostril) of MPXV/USA/luc. Three control mice were inoculated in the same manner with an equal volume of PBS. Bioluminescent imaging was performed using an IVIS 200 series *in vivo* imager (Perkin Elmer, Oakland, CA). On days 19, 21, 23, and 25, two to three mice were sacrificed to collect tissues. Twelve mice were also inoculated intraperitoneally with 10^5 PFU the same virus diluted to 100 microliters of PBS. Three controls were injected intraperitoneally with the same volume of PBS. Two to three mice in this group were sacrificed on days 3, 5, 7, and 9. Although tissues were collected, no further analysis

was performed. Bioluminescent imaging was performed every other day from day 1 until day 29. Luminescence data were analyzed using Living Image software, version 3.2 (Perkin Elmer, Oakland, CA). Region of Interest (ROI) analysis was performed, using rectangular ROIs that covered the mouse body from nose to rump, excluding the tail unless significant luminescence was present on the tail. Luminescence within ROIs was quantified as total flux in photons per second (p/s).

Results

No mice displayed any signs of clinical disease during the study and no weight loss was detected. Luminescence was detected, especially within the IP group, as shown in figures S1 and S2. In the IP group, luminescence continued in the area of the testis in some male mice up to 23 days post-infection. Luminescence spread to the foot in 2 mice, beginning on day 15. In the IN group, luminescence was initially confined to the area of inoculation, but was later detected in the foot and tail of two mice and one mouse, respectively, starting at day 13 post-infection (figures S3 and S4). Figures S5 and S6 show the luminescence of individual mice over time, compared to the average of all controls measured on that day. Data are available at [2]https://doi.org/10.5066/P9S0ABHH.

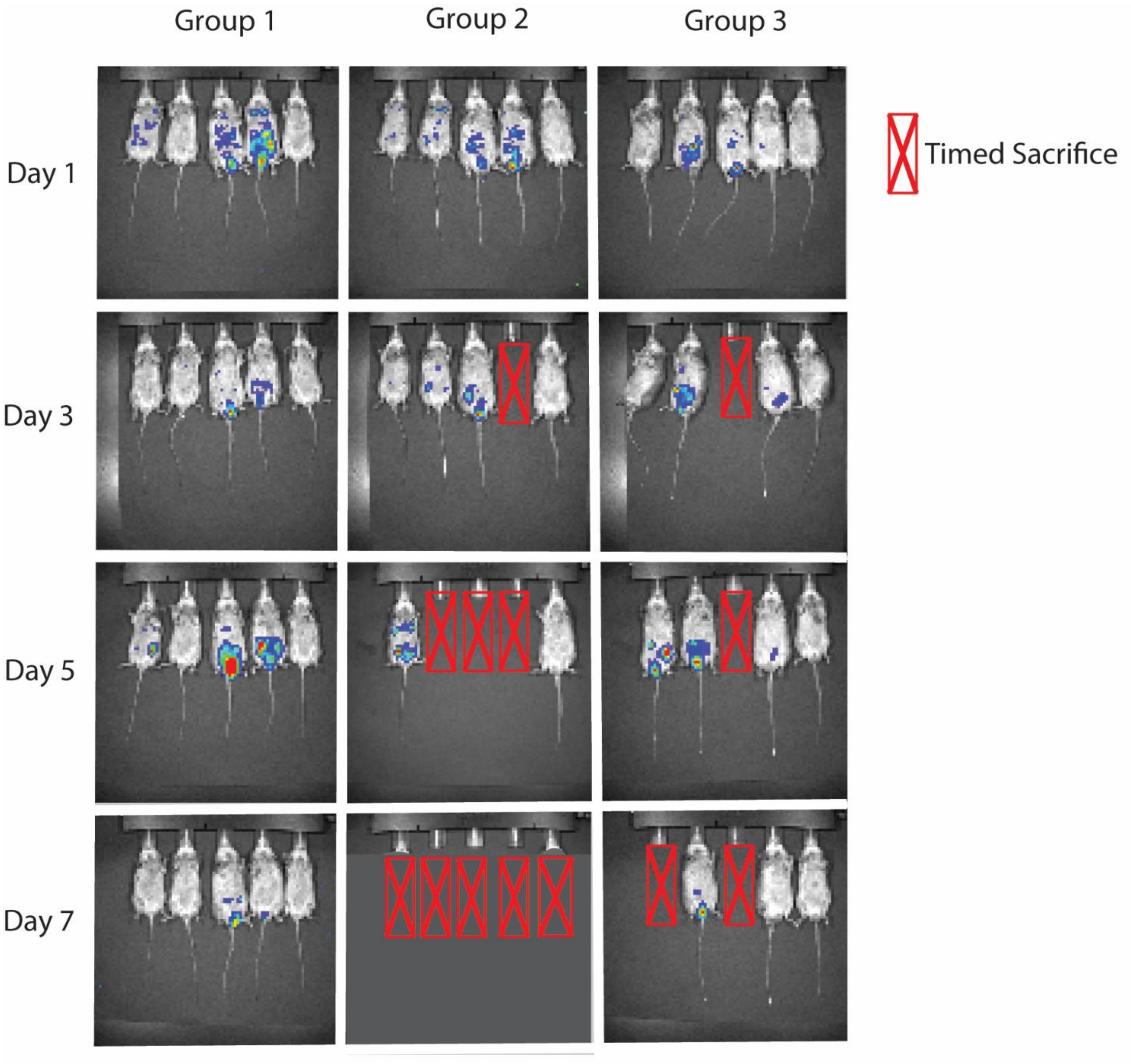

Figure S1. Bioluminescent images of AG129 mice infected with MPXV/USA/Luc intraperitoneally. Luminescence is shown in counts, optimized for each individual image. The color scale is a reverse rainbow scale, with red being the highest output and purple being the lowest output. Images aligned vertically show the same mice over time. The far-right mouse in each image is a control.

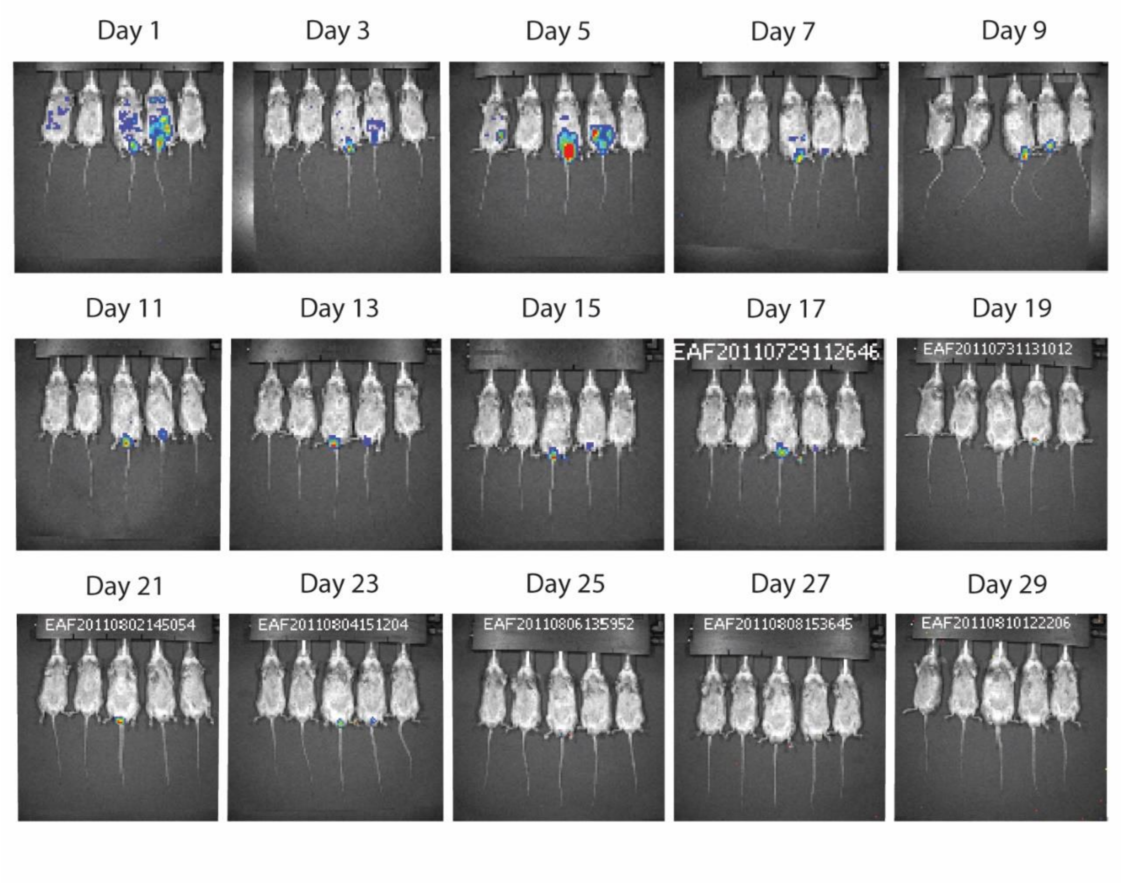

Figure S2. Bioluminescent images of AG129 mice infected with MPXV/USA/Luc intraperitoneally. Luminescence is shown in counts, optimized for each individual image. The color scale is a reverse rainbow scale, with red being the highest output and purple being the lowest output. The mice shown are the 5 mice that were not sacrificed during the entire 29 days. A control mouse in shown on the far right of each image.

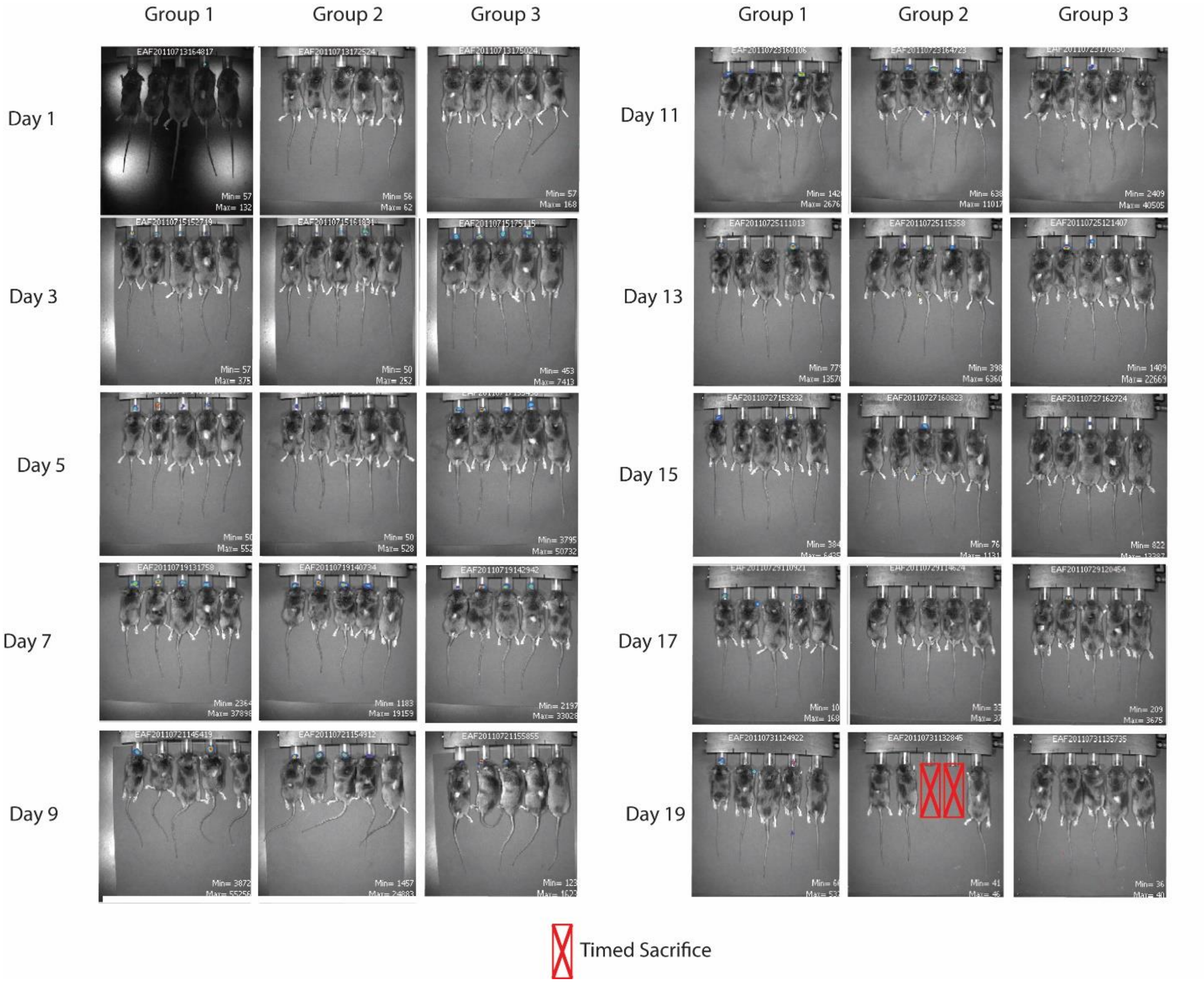

Figure S3. Bioluminescent images of AG129 mice infected with MPXV/USA/Luc intranasally. Luminescence is shown in counts, optimized for each individual image. The color scale is a reverse rainbow scale, with red being the highest output and purple being the lowest output. Images of each group of mice are aligned vertically, such that one can follow individual mice over time. The far-right mouse in each image is a control.

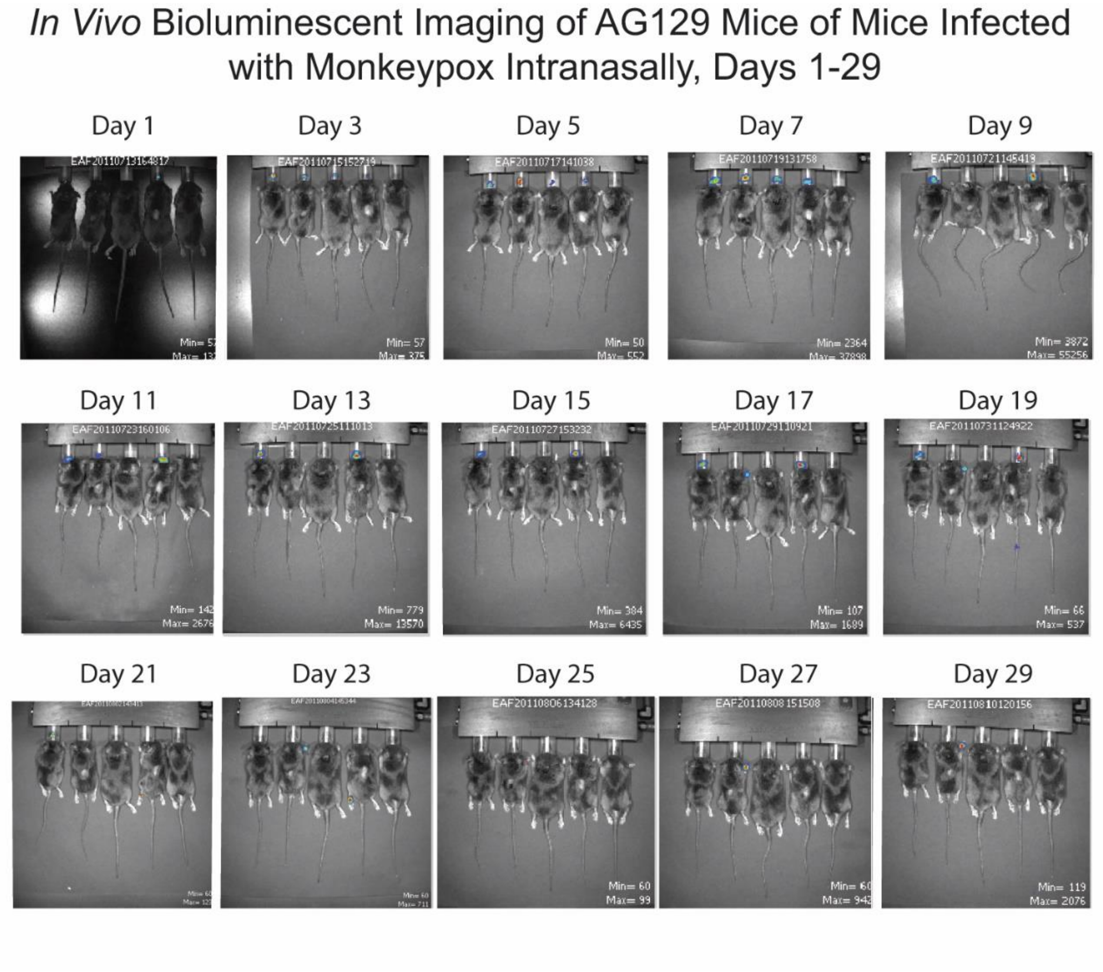

Figure S4. Bioluminescent images of AG129 mice infected with MPXV/USA/Luc intraperitoneally. Luminescence is shown in counts, optimized for each individual image. The color scale is a reverse rainbow scale, with red being the highest output and purple being the lowest output. The mice shown are the 5 mice that were not sacrificed during the entire 29 days. A control mouse in shown on the far-right of each image.

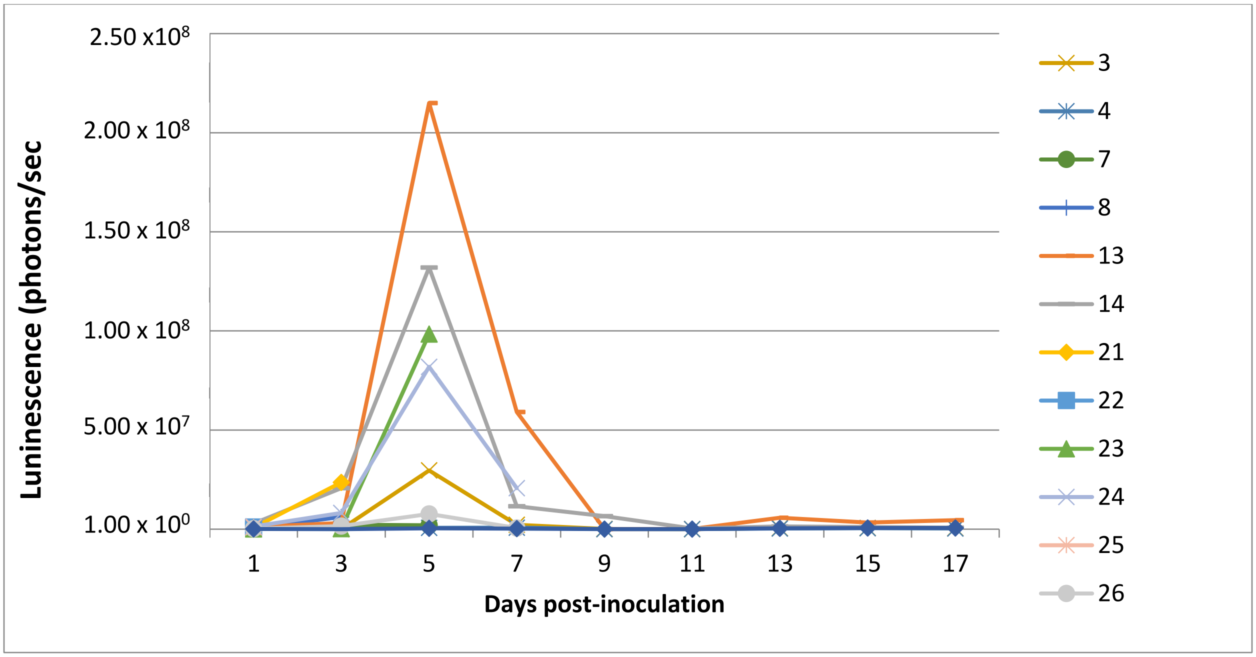

Figure S5. Bioluminescence measured as total flux in photons per second (p/s) of AG129 mice infected intraperitoneally with MPXV/USA/luc. Each line represents a single infected mouse over time (days post-infection). Luminescence of the control mice were averaged and are represented as a single line.

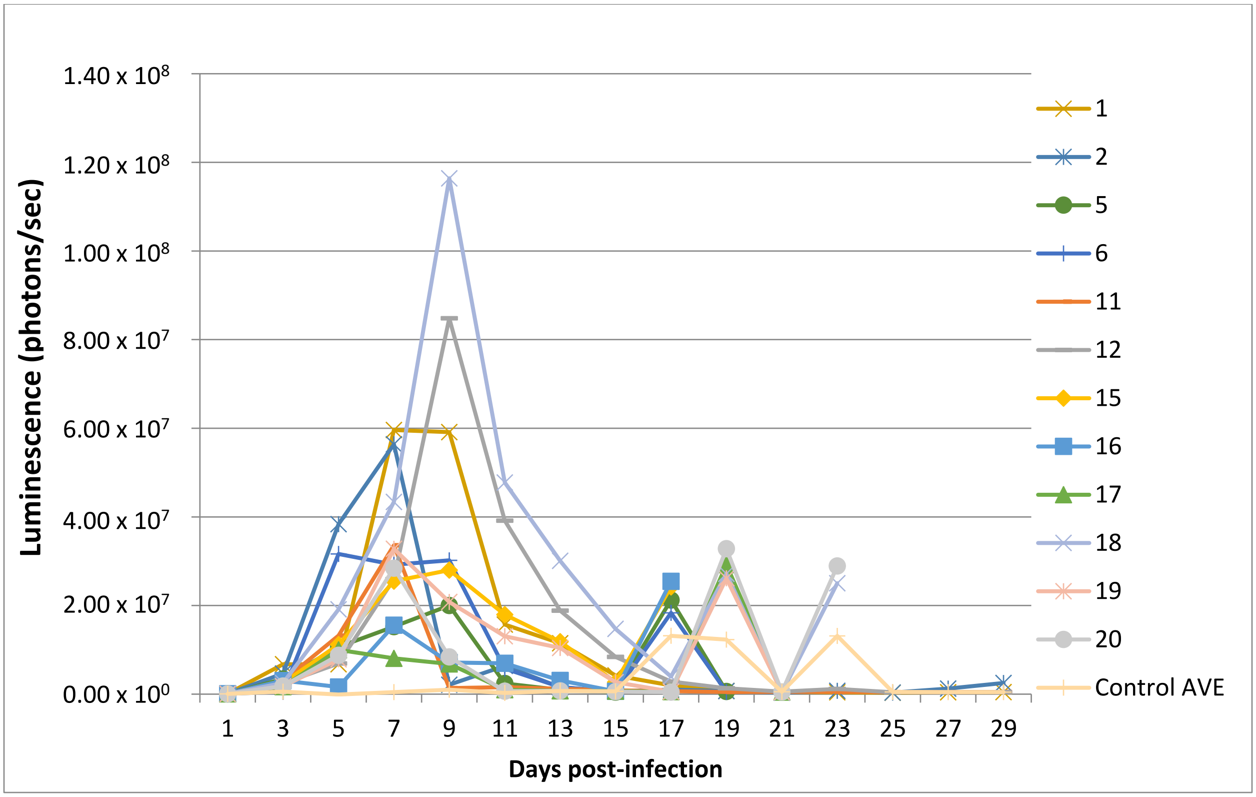

Figure S6. Bioluminescence measured as total flux in photons per second (p/s) of AG129 mice infected intranasally with MPXV/USA/luc. Each line represents a single infected mouse over time (days post-infection). Luminescence of the control mice were averaged and are represented as a single line.

1. Osorio, J.E.; Iams, K.P.; Meteyer, C.U.; Rocke, T.E. Comparison of monkeypox viruses pathogenesis in mice by in vivo imaging. *PloS one* **2009**, *4*, e6592, doi:10.1371/journal.pone.0006592.
2. Falendysz, E.A.; Lopera, J.G.; Rocke, T.E.; Osorio, J.E. Luminescence of AG129 mice infected with recombinant Monkeypox virus expressing firefly luciferase. **2023**.
